# Using the lymph nodal ratio to predict the risk of locoregional recurrence in lymph node-positive breast cancer patients treated with mastectomy without radiation therapy

**DOI:** 10.1186/1748-717X-8-119

**Published:** 2013-05-14

**Authors:** San-Gang Wu, Yong Chen, Jia-Yuan Sun, Feng-Yan Li, Qin Lin, Huan-Xin Lin, Zhen-Yu He

**Affiliations:** 1 Xiamen Cancer Center, Department of Radiation Oncology, the First Affiliated Hospital of Xiamen University, Xiamen, 361003, People’s Republic of China; 2 State Key Laboratory of Oncology in Southern China, Sun Yat-Sen University Cancer Center, Guangzhou 510060, People’s Republic of China; 3 Department of Radiation Oncology, Sun Yat-Sen University Cancer Center, Guangzhou 510060, People’s Republic of China

**Keywords:** Breast cancer, Lymph node ratio, Mastectomy, Recurrences, Radiotherapy

## Abstract

**Background:**

To evaluate the prognostic value of axillary lymph node ratio (LNR) as compared to the number of involved nodes (pN stage) in patients with axillary lymph node-positive breast cancer treated with mastectomy without radiation.

**Methods:**

We performed a retrospective analysis of the clinical data of patients with stage II-III node-positive breast cancer (N=1068) between 1998 and 2007. Locoregional recurrence-free survival (LRFS) and overall survival (OS) were compared based on the LNR and pN staging.

**Results:**

A total of 780 cases were classified as pN1, 183 as pN2, and 105 as pN3. With respect to LNR, 690 cases had a LNR from 0.01-0.20, 269 cases a LNR from 0.21-0.65, and 109 cases a LNR > 0.65. The median follow-up time was 62 months. Univariate analysis showed that both LNR and pN stage were prognostic factors of LRFS and OS (p<0.05). Multivariate analysis indicated that LNR was an independent prognostic factor of LRFS and OS (p<0.05). pN stage had no significant effect on LRFS or OS (p>0.05). In subgroup analysis, the LNR identified groups of patients with different survival rates based on pN stage.

**Conclusions:**

LNR is superior to pN staging as a prognostic factor in lymph node-positive breast cancer after mastectomy, and should be used as one of the indications for adjuvant radiation therapy.

## Introduction

Studies have shown that radiation therapy improves locoregional control of axillary lymph node-positive breast cancer, and thereby benefits survival [1-3]. The positive lymph node status has been used as an indicator for adjuvant radiotherapy after mastectomy [4,5]. However, overall outcomes can be variable depending on the extent of axillary lymph node removal. Additionally, the decision to perform radiation therapy is in part physician dependent.

The lymph node ratio (LNR) is defined as the ratio of the number of positive axillary lymph nodes to the number of removed axillary lymph nodes, and has attracted a great deal of attention. Veronesi et al. [6] has suggested that use of the LNR may minimize the difference between clinical judgment and the real status of the lymph nodes that arises due to differing physician practices. Currently, studies on the LNR have been mainly focused on patients with 1–3 positive nodes [7,8]. The reliability of the LNR in predicting the prognosis in patients with greater than 3 positive nodes has rarely been addressed. In this retrospectively study, we compared the prognostic values of the LNR and number of involved nodes (pN) staging in 1068 patients with axillary lymph node-positive breast cancer without radiation therapy after mastectomy to determine the value of the LNR as an indicator for adjuvant radiation therapy in these patients.

## Materials and methods

### Study population

The study was performed in accordance with the Declaration of Helsinki and was approved by the ethics committee of Sun Yat-Sen University Cancer Center. Written consent was given by the patients for their information to be stored in the hospital database and used for research. A total of 1068 female stage II-III breast cancer patients treated between January 1998 and May 2007 at the Sun Yat-sen University Cancer Center were included in this study. All patients were diagnosed with unilateral breast cancer without initial distant metastasis, and underwent mastectomy and axillary lymph node dissection. Staging was based on the 2009 7th edition of the American Joint Committee on Cancer (AJCC) staging system, and patients with a post-mastectomy pathological stage of T1-4N1-3M0 were included. In all cases, the tumor was completely dissected and surgical margins were negative. No neo-adjuvant therapy was administered before surgery, and no adjuvant radiotherapy was provided after surgery. No patients had any serious comorbid conditions.

### Clinical and pathological factors and lymph node status

Clinical and pathological characteristics were used to assess the risk of locoregional recurrence and death, and included age, menopausal status, T stage, pN stage, and estrogen receptor (ER), progesterone receptor (PR), and human epithelial growth factor receptor family 2 (Her-2) status. T staging and pN staging were determined according to the AJCC staging system (7th edition, 2009). LNR classifications were based on the report by Vinh-Hung et al. [9]. Patients were classified into 3 groups: LNR 0.01-0.20, LNR 0.21 - 0.65, and LNR > 0.65.

### Follow-up and survival endpoints

Follow-up was scheduled every 3–6 months after surgery. Locoregional recurrence-free survival (LRFS) and overall survival (OS) were the primary study endpoints. Locoregional recurrence was defined as pathologically confirmed relapse on the chest wall, supra- and infraclavicular fossa, axillary area, or internal mammary region. Mortality was defined as breast cancer-related death.

### Statistical analysis

Data were analyzed using SPSS 16.0 software. Kaplan-Meier curves were generated to compare the survival rates. The statistical significance of data was analyzed by log-rank test. Cox stepwise regression analysis was used for multivariate analysis, and significant variables in univariate analysis as indicated by p<0.05 were included in the Cox model. Statistical significance was set at p<0.05.

## Results

### Clinical and pathological factors and treatment protocol

A total of 1068 patients with a median age of 47 years (range, 23–90 years) were included in the study. The clinical and pathological characteristics of the patients are summarized in Table 1. The median numbers of axillary lymph nodes removed and positive lymph nodes were 15 (1–45) and 2 (1–44), respectively. Based on the pN staging system, 780 cases (73.1%) were classified as N1, 183 cases (17.1%) as N2, and 105 cases (9.8%) as N3. The median LNR was 0.14 (0.03-1.00). There were 690 cases (64.6%) with a LNR from 0.01-0.20, 269 (25.2%) with a LNR from 0.21-0.65, and 109 (10.2%) with a LNR > 0.65. A total of 1032 patients (96.6%) received chemotherapy following surgery. Among them, 142 cases underwent a regimen consisting of cyclophosphamide, methotrexate, and 5-fluorouracil (CMF), and 890 cases received regimens with anthracycline and/or taxane. The other 36 patients did not receive any chemotherapy. All patients with a positive hormone receptor status were treated with endocrine therapy using tamoxifen and aromatase inhibitors after chemotherapy. Herceptin was used for 4 patients with Her-2 overexpression.

**Table 1:** Patients characteristics and univariate analysis of prognostic factors for survival

**Characteristic**		**LRFS**			**OS**		
	**n**	**5-year (%)**	**10-year (%)**	**p**	**5-year (%)**	**10-year (%)**	**p**
Age (y)							
≤ 35	134	76.0	70.4	0.020^*^	74.4	66.4	0.113
> 35	934	85.4	82.7		80.5	67.4	
Menopausal status							
Premenopausal	697	81.8	79.3	0.026^*^	80.3	68.9	0.589
Postmenopausal	371	89.0	85.1		78.7	63.4	
Tumor size							
T1-2	916	86.4	83.6	<0.001^*^	81.8	70.9	<0.001^*^
T3-4	97	70.2	70.2		61.2	36.1	
Unknown	55						
No of positive lymph nodes							
pN1 (1–3)	780	88.7	86.1	<0.001^*^	86.1	77.7	<0.001^*^
pN2 (4–9)	183	77.0	73.0		69.7	51.0	
pN3 (≥ 10)	105	60.6	53.8		49.9	20.8	
Lymph node ratio							
< 0.20	690	90.2	87.7	<0.001^*^	87.1	78.6	<0.001^*^
0.21-0.65	269	78.6	74.9		75.1	60.9	
> 0.65	109	57.5	52.3		44.3	21.5	
ER status							
Positive	599	85.9	84.4	0.008^*^	83.9	69.0	<0.001^*^
Negative	393	81.0	76.7		72.2	64.2	
Unknown	76						
PR status							
Positive	652	85.9	83.4	0.008^*^	83.0	72.6	<0.001^*^
Negative	340	80.3	77.6		72.3	57.0	
Unknown	76						
HER-2-neu status							
Positive	321	81.8	79.7	0.077	75.6	66.2	0.026^*^
Negative	585	85.5	82.0		82.7	71.2	
Unknown	162						
Chemotherapy regimen							
CMF	142	82.1	79.6	0.332	76.0	60.1	0.069
Anthracycline and/or taxane	850	84.9	81.9		81.5	71.0	
None/unknown	76						
Hormone therapy							
Yes	718	85.7	82.5	0.032^*^	83.7	70.5	<0.001^*^
None	350	81.4	78.8		71.2	60.4	

*LRFS*, Locoregional recurrence-free survival; *OS*, Overall survival; *ER*, Estrogen receptor; *PR*, Progesterone receptor; *Her-2*, Human epithelial growth factor receptor family 2; *CMF*, Cyclophosphamide, methotrexate, and 5-fluorouracil.

^*^p<0.05 indicates a significant difference.

### Survival and disease progression

The median follow-up time for all patients was 62 months (5–154 months). Locoregional recurrence occurred in 155 cases. The 5- and 10-year LRFS rates were 84.3% and 81.3%, respectively. The median time to recurrence was 23 months (range, 4–91 months). Supraclavicular fossa recurrence occurred in 73 cases (47.1%), chest wall recurrence in 47 cases (30.3%), axillary lymph node recurrence in 3 cases (1.9%), internal mammary recurrence in 2 cases (1.3%), and infraclavicular fossa recurrence in 2 cases (1.3%). There were 28 cases in which recurrence occurred at ≥ 2 sites (18.1%). A total of 240 patients died, among whom 229 died as a result of breast cancer and 11 died of other disorders. The 5- and 10-year OS rates were 79.7% and 67.3%, respectively.

### Analysis of prognostic factors

**Figure 1:**
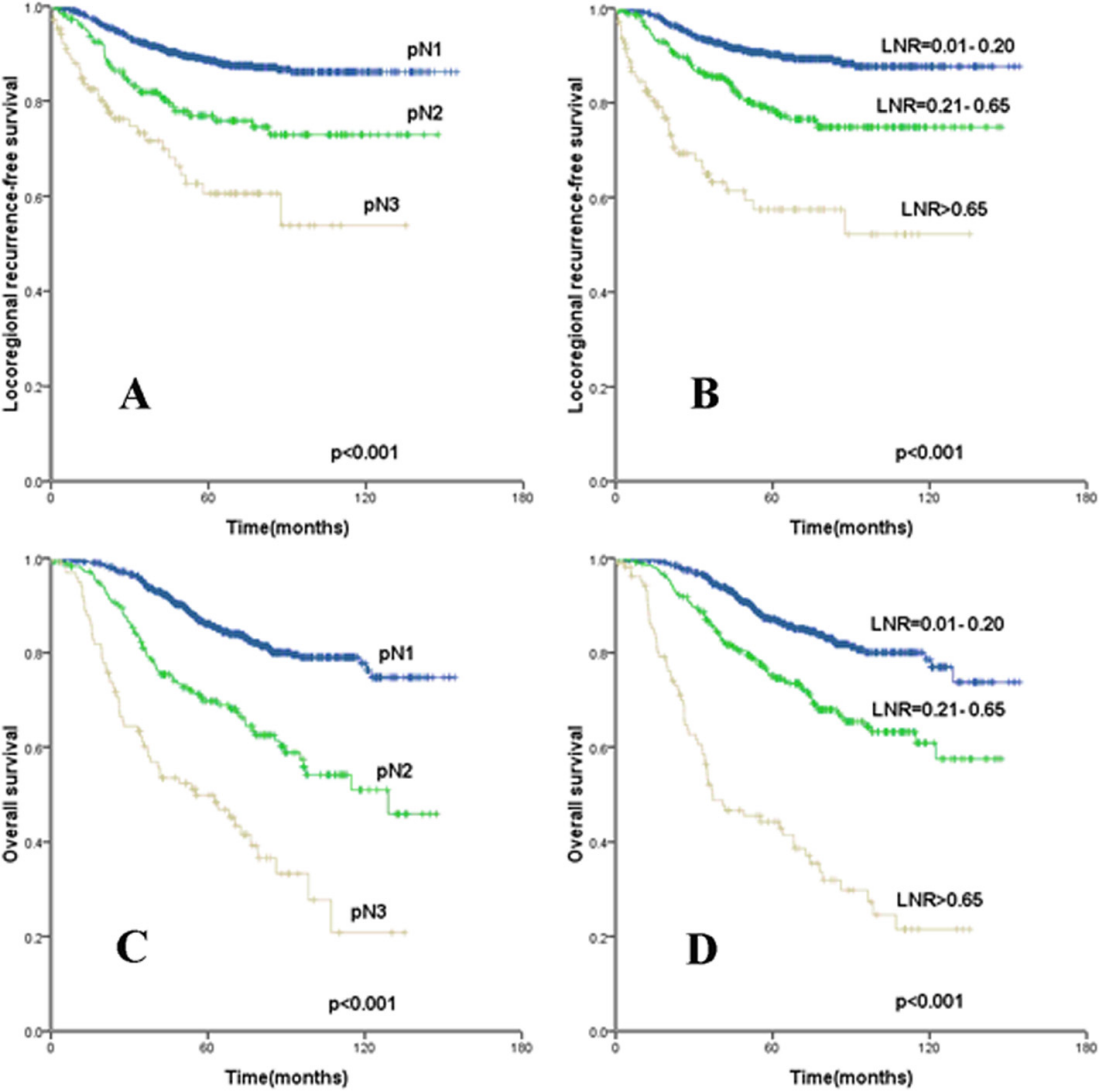
**Kaplan-Meier cumulative survival curves for different lymph node ratio (LNR) and pN stage. **(**A**, **B**) Locoregional recurrence-free survival and (**C**, **D**) overall survival for (**A**, **C**) pN and (**B**, **D**) LNR.

Univariate analysis showed that age, menopausal status, T stage, pN stage, LNR, ER and PR status, and hormone therapy were all prognostic factors of LRFS (p<0.05 ). The 10-year LRFS rates were 86.1%, 73.0%, and 53.8% for stage pN1, pN2, and pN3, respectively (p<0.001), while the rates were 87.7%, 74.9%, and 52.3% for a LNR of 0.01-0.20, LNR of 0.21 - 0.65, and a LNR > 0.65, respectively (p<0.001). T stage, pN stage, LNR, ER, PR, and Her-2 status, and hormone therapy were all prognostic factors of OS (p< 0.0 5) (Table 1 and Figure 1).

The variables demonstrating statistical significance by univariate analysis were further analyzed using multivariate analysis in the Cox regression model. When pN and LNR were included as covariants, the LNR remained an independent prognostic factor for LRFS and OS (p<0.05), with a higher LNR indicating a higher risk, but pN stage exhibited no effect on LRFS and OS (p>0.0 5) (Table 2).

**Table 2:** Multivariate analysis of survival by lymph node ratio and pN stage

**Characteristic**	**LRFS**		**OS**	
	**HR (95% CI)**	**p**	**HR (95% CI)**	**p**
Age (y)				
≤ 35 vs. > 35	1.664 (1.082-2.558)	0.020^*^	—	
Menopausal status				
Pre- vs. postmenopausal	1.623 (1.106-2.383)	0.105	—	
Tumor size				
T3-4 vs. T1-2	1.636 (1.029-2.600)	0.037	1.392 (0.917-2.113)	0.121
ER status				
Negative vs. positive	1.536 (1.095-2.155)	0.013^*^	1.054 (0.699-1.590)	0.802
PR status				
Negative vs. positive	1.626 (1.148-2.302)	0.317	1.490 (1.040-2.136)	0.030^*^
HER-2-neu status				
Positive vs. negative	—		1.208 (0.888-1.644)	0.228
Hormone therapy				
None vs. yes	1.180 (0.731-1.904)	0.498	1.570(1.093-2.255)	0.015^*^
Lymph node ratio				
< 0.20	1 (Reference)		1 (Reference)	
0.21-0.65	1.886 (1.273-2.794)	0.002^*^	1.964 (1.387-2.782)	<0.001^*^
> 0.65	5.013 (3.191-7.877)	<0.001^*^	7.381 (5.161-10.557)	<0.001^*^
Number of positive lymph nodes				
pN1	1 (Reference)			
pN2	0.828 (0.476-1.442)	0.522	1.327 (0.808-2.179)	0.263
pN3	0.894 (0.437-1.828)	0.907	1.654 (0.904-3.027)	0.103

*LRFS*, Locoregional recurrence-free survival; *OS*, Overall survival; *ER*, Estrogen receptor; *PR*, Progesterone receptor; *Her-2*, Human epithelial growth factor receptor family 2; *HR*, Hazard ratio; *CI*, Confidence interval.

^*^p<0.05 indicates a significant difference.

### Prognostic significance of LNR based on pN stage

**Figure 2:**
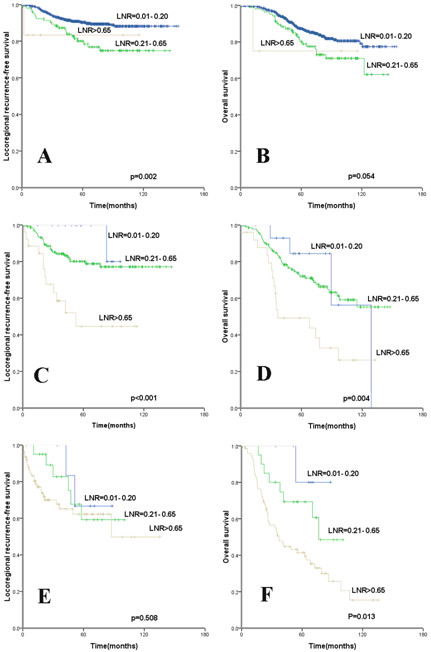
**Kaplan-Meier cumulative survival curves for different lymph node ratio according to different pN stage. **(**A**, **C**, **E**) Locoregional recurrence-free survival (LRFS) and (**B**, **D**, **F**) overall survival for (**A**, **B**) pN1, and (**C**, **D**) pN2, and (**E**, **F**) pN3.

The subgroup analysis of the prognostic significance of LNR according to different pN stages is shown in Table 3 and Figure 2. For pN1 patients, the 5-year and 10-year LRFS for a LNR of 0.21-0.65 were 80.4% and 74.8%, respectively, which were significantly lower than values of 90.3% and 88.2%, respectively, when the LNR was < 0.20 (p 0.002). The 5-year LRFS for pN2 patients with a LNR of 0.01-0.20, 0.21 - 0.65, and > 0.65, were 80.0%, 82.1%, and 44.6%, respectively (p<0.001). Among pN3 patients, the LNR had no impact the prognosis, but the LRFS was lower, the 5-year LRFS was 66.7%, 59.2%, and 66.2% for patients with a LNR of 0.01-0.20, 0.21 - 0.65, and > 0.65, respectively (p=0.508). The LNR was a prognosis factor with respect to OS based on pN stage.

**Table 3:** Impact of lymph node ratio according to different pN stages

**Characteristic**		**LRFS**			**OS**		
	**n**	**5-year (%)**	**10-year (%)**	**p**	**5-year (%)**	**10-year (%)**	**p**
pN1 (1–3 positive nodes)							
Lymph node ratio							
< 0.20	667	90.3	88.2	0.002^*^	87.3	79.0	0.054
0.21-0.65	107	80.0	74.8		80.1	71.0	
> 0.65	6	83.3	—		75.0	—	
pN2 (4–9 positive nodes)							
Lymph node ratio							
< 0.20	16	80.0	—	<0.001^*^	84.4	56.3	0.004^*^
0.21-0.65	141	82.1	77.2		72.0	55.2	
> 0.65	26	44.6	—		49.2	26.2	
pN3 (≥ 10 positive nodes)							
Lymph node ratio							
< 0.20	7	66.7	—	0.508	80.0	—	0.013^*^
0.21-0.65	21	59.2	—		69.3	—	
> 0.65	77	62.2	49.8		41.4	15.4	

*LRFS*, Locoregional recurrence-free survival; *OS*, Overall survival.

^*^p<0.05 indicates a significant difference.

## Discussion

Although significant progress has been made in understanding molecular biomarkers of breast cancer, axillary lymph node status remains one of the fundamental prognostic factors that guides the decision for post-mastectomy radiation therapy [4,5]. Presently, the tumor, regional lymph node, metastasis (TNM) staging system, established by the AJCC, is accepted and utilized worldwide. In this system, the pN staging of axillary lymph nodes is based on the absolute number of lymph nodes. Although easy to use, the accuracy of the approach may be affected by the number of removed axillary lymph nodes, and thus may be subject to unintended variability. In the present study, we explored the prognostic value of LNR in stage II-III node-positive breast cancer patients without radiotherapy after mastectomy, and demonstrated that the LNR can better predict tumor recurrence and mortality.

The 82b and 82c randomized trials of radiotherapy conducted by the Danish Breast Cancer Cooperative Group provided evidence that postoperative adjuvant radiotherapy has therapeutic value in axillary lymph node-positive breast cancer; however, the median number lymph nodes removed was 7, suggesting that adjuvant therapy used at that time appears insufficient [10]. Nagao et al. [11] showed that the LRFS rate was 8.7% with radiotherapy and 7.3% without radiotherapy after mastectomy in lymph node-positive nodes patients, with a median number of lymph nodes removed of 18.6. In a study by Gentilini et al. [12] in which patients received no radiotherapy after mastectomy plus total axillary clearance (I/II/III region) where the median number of lymph nodes removed was 23, with the 5-year LRFS rates were 3.0%, 8.1%, and 9.9% for N0, N1 and ≥ N2 stage disease, respectively. These studies suggest that sufficient clearance of lymph nodes is helpful to reduce locoregional recurrence, and thereby affects the decision to administer adjuvant radiotherapy.

Physician differences and experience can affect the accuracy of pN staging. Due to variations in the clearance of axillary lymph nodes and different physical examination findings, the accuracy of pN staging can be compromised. Furthermore, the optimal number of the axillary lymph nodes that need to be removed remains controversial [13,14]. However, Fisher et al. [7] demonstrated that use of the LNR may minimize the difference in prognosis seen among hospitals due to different degrees of lymph node clearance. Several studies have shown that use of the LNR may change pN staging based on the AJCC system, and should be considered in addition to TNM staging in order to provide better guidance regarding adjuvant therapy [7,9,15-19]. Our study suggests that the LNR is an independent prognostic factor of LRFS and OS, and pN staging lost significance when LNR was included in the multivariate analysis. This suggests that LNR has better prognostic value than pN staging.

Locoregional recurrence is a determinant in the selection of adjuvant radiotherapy after mastectomy. The St. Gallen Breast Cancer Conference in 1998 recommended postoperative radiotherapy when the expected LRFS rate was > 20% [20]. In recent years, research has been concentrated on the effect of LNR on OS in patients with breast cancer [15-17], and the studies on LRFS have been mainly conducted in patients with 1–3 metastatic lymph nodes (pN1) [7,8]. Our study suggests that LNR is superior to pN staging as a predictor of LRFS in patients with breast cancer. We performed subgroup analysis of the prognostic value of LNR according to different pN stages, and for pN1 patients the 5-year and 10-year locoregional recurrence rates (LRR) were 20% and 25.2%, and the LRR was above 20% in stage pN2 and pN3 patients across all LNR groups. The value of adjuvant radiotherapy in 1–3 metastatic lymph nodes is still controversial. Therefore, we recommend that LNR should be employed as one of the indications for adjuvant radiotherapy after mastectomy (i.e., radiation therapy should be considered if the LNR is > 0.20), instead of a treatment decision based only on pN stage.

Our study does have several limitations. First, the conclusions are based on a single center retrospective study and may not be generalizable to other populations. However, an increasing amount of data now supports the prognostic value of LNR in breast cancer, e.g., the International Nodal Ratio Working Group is currently undertaking research to establish the prognostic significance of LNR in breast cancer [19]. Second, there is no consensus on standard cutoff points for LNR [7,9,18,19]. The LNR cutoff points used in this study were based on the report by Vinh-Hung et al., which has been validated in studies in other countries [15-17]. Our study suggests that the cutoff points for the LNR used by Vinh-Hung et al. are applicable to Chinese women with breast cancer, but a larger sample size is needed to confirm the results. Third, the prognostic value of Ki-67 was not analyzed due to missing data, and most of the patients did not received trastuzumab therapy.

## Conclusions

In summary, our study demonstrated that LNR is a better prognostic predictor than pN stage in patients with axillary lymph node-positive breast cancer after mastectomy, and should be used as one of the indications for adjuvant radiation therapy . Further prospective studies are necessary to assess the impact of LNR on prognosis, and to define the usefulness of postoperative radiotherapy.

## Abbreviations

LNR: Lymph node ratio; ER: Estrogen receptor; PR: Progesterone receptor; Her-2: Human epithelial growth factor receptor family 2; LRFS: Locoregional recurrence-free survival; OS: Overall survival; CMF: Cyclophosphamide, methotrexate, and 5-fluorouracil; AJCC: American Joint Committee on Cancer; TNM: Tumor, regional lymph node, metastasis.

## Competing interests

The authors declare they have no competing interests of the article.

## Authors’ contributions

SGW,YC, and JYS carried out the data collection and writing of the manuscript; SGW helped to conceive the study; ZYH contributed to the design of the study; FYL and QL helped to collect data, HXL participated in statistical analysis. All authors read and approved the final manuscript.
